# Type I Interferon Reaction to Viral Infection in Interferon-Competent, Immortalized Cell Lines from the African Fruit Bat *Eidolon helvum*

**DOI:** 10.1371/journal.pone.0028131

**Published:** 2011-11-30

**Authors:** Susanne E. Biesold, Daniel Ritz, Florian Gloza-Rausch, Robert Wollny, Jan Felix Drexler, Victor M. Corman, Elisabeth K. V. Kalko, Samuel Oppong, Christian Drosten, Marcel A. Müller

**Affiliations:** 1 Institute of Virology, University of Bonn Medical Centre, Bonn, Germany; 2 Noctalis, Centre for Bat Protection and Information, Bad Segeberg, Germany; 3 Institute of Experimental Ecology, University of Ulm, Ulm, Germany; 4 Smithsonian Tropical Research Institute, Balboa, Panama; 5 Kwame Nkrumah University of Science and Technology, Kumasi, Ghana; Veterinary Laboratories Agency, United Kingdom

## Abstract

Bats harbor several highly pathogenic zoonotic viruses including Rabies, Marburg, and henipaviruses, without overt clinical symptoms in the animals. It has been suspected that bats might have evolved particularly effective mechanisms to suppress viral replication. Here, we investigated interferon (IFN) response, -induction, -secretion and -signaling in epithelial-like cells of the relevant and abundant African fruit bat species, *Eidolon helvum (E. helvum)*. Immortalized cell lines were generated; their potential to induce and react on IFN was confirmed, and biological assays were adapted to application in bat cell cultures, enabling comparison of landmark IFN properties with that of common mammalian cell lines. *E. helvum* cells were fully capable of reacting to viral and artificial IFN stimuli. *E. helvum* cells showed highest IFN mRNA induction, highly productive IFN protein secretion, and evidence of efficient IFN stimulated gene induction. In an Alphavirus infection model, O'nyong-nyong virus exhibited strong IFN induction but evaded the IFN response by translational rather than transcriptional shutoff, similar to other Alphavirus infections. These novel IFN-competent cell lines will allow comparative research on zoonotic, bat-borne viruses in order to model mechanisms of viral maintenance and emergence in bat reservoirs.

## Introduction

The order chiroptera (bats) is one of the most diverse and geographically wide-spread orders within the mammals constituting 20% of all mammalian species [1]. Chiroptera are subdivided into two suborders Yango- and Yinpterochiroptera. The latter includes frugivorous/nectarivorous bats (flying foxes) with species like *Rousettus aegyptiacus* (*R. aegyptiacus*), *Pteropus alecto* (*P. alecto*) and *Eidolon helvum* (*E. helvum*) as well as the insectivorous bat *Rhinolophus cf. landeri* (*R. cf. landeri*, Rhinolophidae). Yangochiroptera comprise bats from the three superfamilies Emballonuroidea, Noctilionoidea, Vespertilionoidea including the insectivorous species *Myotis daubentonii* (*M. daubentonii*), *Pipistrellus spec.* (both Vespertilionidae), *Tadarida brasiliensis* (*T. brasiliensis*, Molossidae), *Hipposideros cf. caffer/ruber* (*H. cf. caffer/ruber*, Hipposideridae) [2], [3]. Bats have been shown to host relevant human pathogens like Rabies-, Ebola-, Marburg-, henipaviruses (Hendra-, Nipah-) and severe acute respiratory syndrome (SARS)-like Coronaviruses [4]. The ability to control such highly pathogenic viruses raises the question whether bats might have evolved particularly effective mechanisms of immune control [5]–[7]. Little is known about the immune system of bats. It has been shown that bats develop immunoglobulins after infection and have lymphoid development similar to that in other mammals [8]–[10]. However, information on the innate immune response of bats is particularly scarce. Toll-like receptor genes have been identified in *R. leschenaulti* and *P. alecto* [11], [12]. Cells from *Pteropus* species have been shown to produce high amounts of interferon (IFN)-λ after stimulation with the double-strand (ds)RNA analogue poly IC, and after infection with the bat-associated paramyxovirus, Tioman [13]. Conversely, infection with the highly pathogenic paramyxovirus Hendra virus resulted in no induction of IFN expression and concomitant inhibition of IFN signaling, suggesting the presence of specific viral IFN antagonists [14]. A conserved functionality of IFN signaling in different mammalian cell cultures including epithelial lung cells from *Tadarida brasiliensis* (Tb1-Lu) was already described earlier [15], [16]. However, there remains a fundamental lack of knowledge on the ways type I IFNs are induced and IFN signals are processed in bat cells.

Because type I IFN is a major barrier towards virus infection, quantitative comparisons between different mammalian systems are of particular interest. Currently there are hardly any bat cell lines available whose fundamental properties in IFN induction and -response have been characterized in a comparative manner. Here we present a set of essential tools to characterize IFN induction and -response in bat cells, and introduce a novel group of highly IFN-competent, immortalized bat cell lines from the species *E. helvum* that hosts relevant zoonotic viruses including Henipa- and Lyssaviruses [17], [18]. We compare paramount patterns of IFN induction and response in these *E. helvum* cells with that in prototype murine and primate cell lines.

## Methods

### Ethics statement

For all capturing and sampling, permission was obtained from the Wildlife Division, Forestry Commission, Accra, Ghana. Samples were exported under a state contract between the Republic of Ghana and the Federal Republic of Germany, and under an additional export permission from the Veterinary Services of the Ghana Ministry of Food and Agriculture (permit no. CHRPE49/09; A04957).

### Cell culture

All cells were cultivated in DMEM (Dulbecco's Modified Eagles Medium) (PAA, Cölbe, Germany) with 4.5 g/L Glucose (PAA), supplemented with 10% Fetal Bovine Serum (PAA), 1% Penicillin/Streptomycin 100× concentrate (Penicillin 10000 units/ml, Streptomycin 10 mg/mL) (Life Technology), 1% L-Glutamine 200 mM, 1% Sodium Pyruvate 100 mM (PAA), 1% MEM nonessential amino acids (NEAA) 100× concentrate (PAA). Cells were generally incubated at 37°C and 5% CO_2_. As prototype mammalian cells we applied simian virus (SV) 40 large T antigen immortalized mouse embryonic fibroblasts (MEF) generated in-house from 129/SvJ mice [19], African green monkey kidney cells (MA104, kindly provided by Friedemann Weber, University of Marburg) and human lung adenocarcinoma epithelial cell line (A549, CCL-185). For titration of O'nyong nyong virus (ONNV) Vero E6 cells (ATCC CRL-1586) were used.

Under the auspices of Ghana authorities bats were caught with mist nets, anaesthetized with a Ketamine/Xylazine mixture and euthanized to perform organ preparations (permit no. CHRPE49/09; A04957). Organs from *E. helvum* (embryo kidney and lung), *R. aegyptiacus* (kidney), *M. daubentonii* (lung), *Pipistrellus spec.* (kidney), *H. cf. caffer/ruber* (embryo) and *R. cf. landeri* (kidney) were minced, trypsinized, and cultured in DMEM medium by titration and seeding at 1∶100 dilution in cell culture flasks as described previously [20]. Imipenem (Zienam, MSD, Haar, Germany) and Amphotericin B (PAA) were added to minimize contamination risks. Immortalization was done by lentiviral transduction of the large T antigen of SV40. Immortalized cells were expanded and stock frozen or processed further for subcloning. All cell cultures were genotyped by amplification of mitochondrial cytochrome b as previously described using primers L14724 and H15149 (**Table S1**) [21], [22] and were controlled for mycoplasma [23], SV 5 (in-house assay, **Table S1**), lyssaviruses [24] and filoviruses [25] by RT-PCR.

### Nucleic acid extraction and real-time RT-PCR

Viral RNA was extracted from cell culture supernatant with the QIAamp Viral RNA mini Kit (QIAGEN, Hilden, Germany). Total RNA from 90% confluent cells was isolated using the RNeasy Mini kit (QIAGEN) and reverse-transcribed with random hexamer primers (Life Technologies, Karlsruhe, Germany). Fragments of target genes were amplified from cDNA by low-stringency PCR. After initial denaturation for 2 min at 94°C, touchdown PCR was done for 10 cycles (94°C/20 s, 66-56°C, delta 1°C/10 s, 72°C/60 s). The remaining 30 PCR cycles included 94°C/20 s, 56°C/20 s, 72°C/60 s. Fragments were sequenced and used to design real-time RT-PCR assays targeting genes relevant for the IFN system as well as reference genes, in domains conserved among bat species (**Table S1**). Probes were tagged with 5′-carboxyfluorescein and 3′-black hole quencher (Biomers, Ulm, Germany). Real-time RT-PCR was processed using the LightCycler® 480 Real-Time PCR System (Roche, Basel, Switzerland).

For quantification of ONNV genome equivalents (GE) the dilution end-point was defined as one PCR unit. Log PCR units per ml for each experimental sample were calculated from the linear equations of the dilution series [26]. To determine the fold-induction of the different target genes (*IFN*, *MxA*, *ISG56*) the 2^−ΔΔCt^ method was applied with TATA-box binding protein (TBP) as housekeeping gene [27].

### Virus infection and plaque titration

ONNV infections were performed at multiplicity of infection (MOI) of 2.5 and 0.0025 respectively. Virus was diluted in serum-free medium Optipro (Life Technologies). Cells were inoculated for 1 h at 37°C and washed twice with PBS after infection. Samples were taken at time points 0, 8 and 24 h post infection (hpi). Titration of ONNV was performed by plaque assay as previously described [26], [28]. Vero E6 cells were seeded in 24-well plates at a density of 4×10^5^ cells per ml. Virus supernatant was added and after 1 h incubation the inoculum was removed and cells were washed with PBS (PAA). 2× MEM (Biochrom, Berlin, Germany), supplemented with 0.44% (w/v) NaHCO_3_ (Roth, Karlsruhe, Germany), 20% FCS (PAA) and 2% Penicillin/Streptomycin, was mixed in a 1∶1 ratio with 2.4% Avicel (FMC, BioPolymers, Brussels, Belgium) and 0.5 ml per well was added. After 2 days the overlay was discarded and cells were stained with a 0.2% crystal violet and 20% ethanol (Roth) containing solution.

### IFN induction by infection and transfection

Rift valley fever virus clone 13 (RVFV 13) infection for IFN induction was done as described above by infecting cells at an MOI of 1. After 24 h, all supernatants were harvested and virus was inactivated by β-propiolactone (β-PL, Ferak Berlin, Germany) treatment as described below. IFN stimulation by poly IC was performed by transfection of 8×10^5^ cells per 6-well with 5 µg poly IC using Nanofectin (PAA) according to the manual instructions.

### Virus inactivation

RVFV 13- and ONNV-containing supernatants were inactivated with β-PL as previously described [29]–[31]. Briefly, the supernatants were collected and RVFV 13 was inactivated by incubating with 0.05% of β-PL at 4°C for 16 h. The hydrolysis of β-PL was achieved by incubation at 37°C for 2 h. For ONNV a final concentration of 0.1% of β-PL was needed for total inactivation. All β-PL treated samples were subjected to a virus plaque assay to control complete inactivation. To ensure equal treatment of the samples, supernatants of negative controls and cells stimulated with poly IC were also treated with β-PL.

### Vesicular stomatitis virus (VSV) bioassay

A classical VSV bioassay was performed as described previously [32]. EidNi/41.3, MEF and MA104 cells were seeded in 12-well plates at a density of 4×10^5^ cells per ml. After 24 h the inactivated supernatants or a pan-species IFN-α (pan-IFN, a universal recombinant type I human IFN-α A/D hybrid, PBL Biomedical Laboratories/Axxora, Lörrach, Germany) standard dilution series were added to the respective cell lines. An internal standard curve comprising five concentrations (0.5, 1, 10, 20 and 40 units/ml) of pan-IFN diluted in medium of mock treated cells was applied in every experiment. The external pan-IFN standard curves for the EC_50_ calculation were performed in quadruplicates and comprised pan-IFN concentrations between 0.25 and 150 units/ml diluted in DMEM. 24 h posttreatment, IFN containing supernatants were removed and cells were washed with PBS. The cells were then infected with VSV at an MOI of 0.025. After 1 h of virus adsorption cells were washed again and overlayed with Avicel as described above. After 2 days the overlay was discarded, the cells were fixed and stained as before.

### Calculation of EC_50_ values and determination of normalized IFN concentrations

The amount of plaques of the applied pan-species IFN standard curves or of the test samples was calculated in percentage (maximal plaque count from the negative control was set to 100%). The equation of the internal standard was used to calculate IFN amounts in the samples. EC_50_ values were defined as IFN concentrations which reduced the number of plaques to 50%. Afterwards the values were multiplied by the dilution factors and figures were normalized to the amount of IFN per ml. To define comparable, species-independent IFN concentrations the calculated amount of IFN was divided by the EC_50_ of each internal standard curve. Thus, the normalized amount of IFN could directly be compared.

### SDS-PAGE and Western blot analysis

Protein analysis was essentially done as described elsewhere [33]. Generally, cells were lysed in RIPA lysis buffer (150 mM NaCl, 1% Igepal CA-630, 0.5% sodium deoxycholat, 0.1% SDS, 50 mM Tris (pH 8.0), protease inhibitor cocktail III, Benzonase 25 units/ml, 5 mM dithiothreitol (Merck, Darmstadt, Germany)) and separated on a 12% SDS-PAGE gel. Western blotting was performed by using mouse-anti-Mx1/2/3 (C-1, Santa Cruz, Heidelberg, Germany), goat-anti-IFIT/ISG56 (L-16, Santa Cruz), mouse-anti-actin (Sigma) immunoglobulins at dilutions 1∶1000. Secondary detection was done with the help of horseradish peroxidase labeled goat-anti-mouse and rabbit-anti-goat antibody (1∶20000, Dianova, Hamburg, Germany) and SuperSignal® West Femto Chemiluminescence Substrate (Thermo Fisher Scientific, Bonn, Germany).

## Results and Discussion

### Generation of cell cultures from an Old World fruit bat

**Figure 1:**
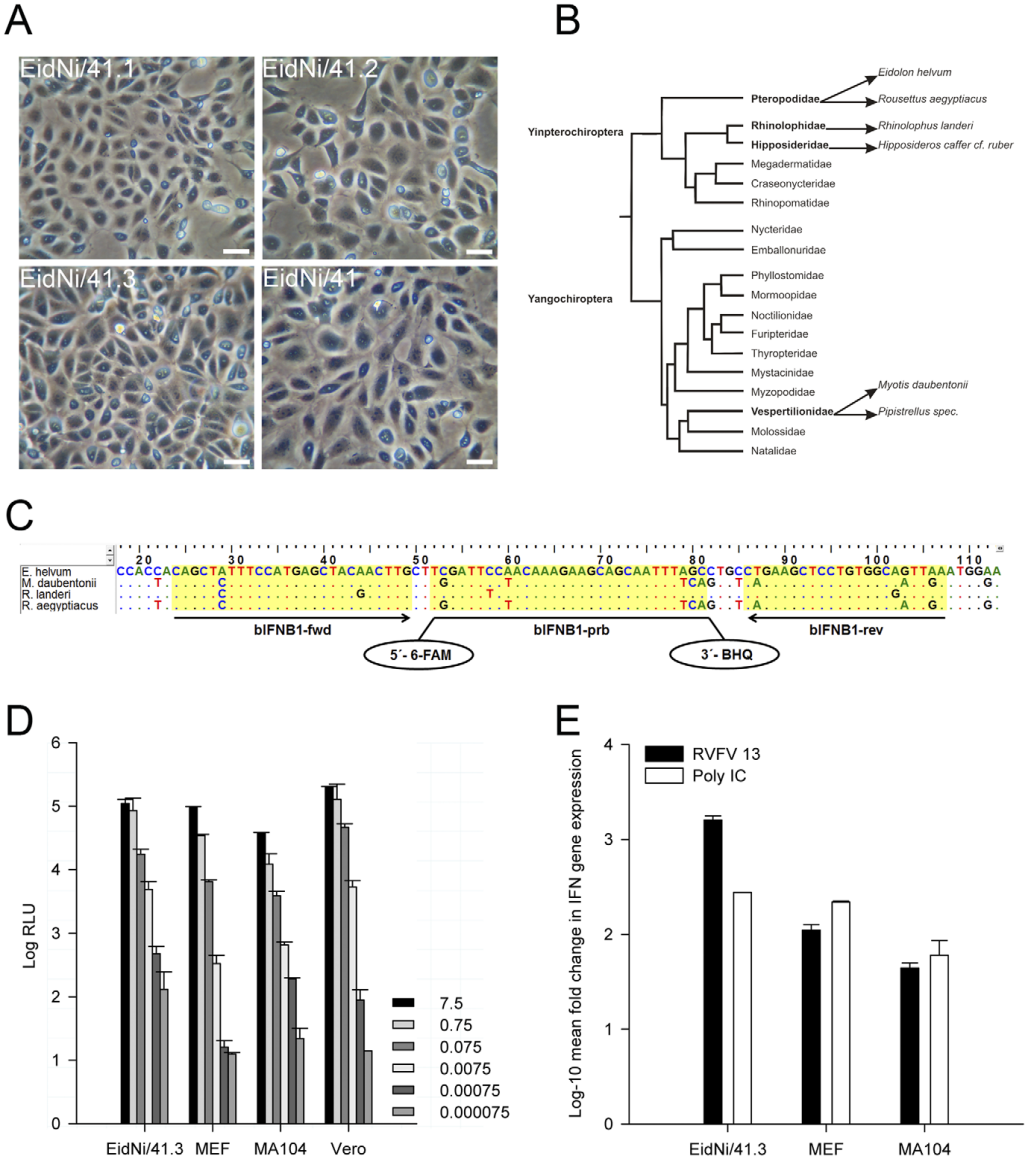
Generating immortalized bat cell cultures and measuring interferon (IFN)-β mRNA induction. (A) Primary bat cell cultures were generated from an embryonic kidney of *E. helvum*. Cell cultures were immortalized by lentiviral transduction of a simian virus 40 large T antigen. Three clonal cell lines (EidNi/41.1, 41.2 and 41.3) were prepared from a mixed culture (EidNi/41) by end point dilution plating. Bars indicate 20 µm. (B) Phylogenetic relationships of different selected bat species for the generation of target gene sequences (adapted from [3]). For the IFN-β gene four species were selected. (C) Alignment of bat IFN-β genes (*E. helvum*, *M. daubentonii*, *R. cf. landeri*, *R. aegyptiacus*) and positions of primers and probe for a pan-bat real-time RT-PCR. (D) Replication of a *Renilla* luciferase expressing Rift valley fever virus clone 13 (RVFV 13) in various cell lines (EidNi/41.3, MEF (mouse embryonic fibroblasts), MA104 (African green monkey kidney) and as reference Vero cells at different MOIs. Cells were infected at different ten-fold diluted MOIs (7.5 until 0.000075) and lysed 24 hpi with *Renilla* lysis buffer (Promega). Replication was detected by *Renilla* luciferase read-out. Experiments were performed in duplicates. Highest replication was found in Vero cells followed by EidNi/41.3, MEF, MA104 (between10 to 100-fold less compared to Vero cells). (E) IFN-β mRNA transcription was induced by either RVFV 13 infection (MOI 1) or poly IC transfection (5 µg per 6-well). IFN-β and TATA-box binding protein (housekeeping gene) mRNA was quantified by species-specific real-time RT-PCR assays. The fold induction was calculated with the 2^−ΔΔCt^ method.

Old World frugivorous/nectarivorous bats or flying foxes (Pteropodidae; formerly classified as “Megachiroptera”) comprise species like *R. aegyptiacus* and *E. helvum*. Whereas *R. aegyptiacus* is a known reservoir for Marburg virus [34]–[36], *E. helvum* was shown to carry Henipa-like viruses [18] and Lagos bat virus [17]. Only for *R. aegyptiacus* [37] and *Tadarida brasiliensis* (ATCC: CCL-88) cell cultures are commercially available. *E. helvum* bats roosting in Kumasi, Ghana, were investigated shortly before the parturition season in 2009. One pregnant female was euthanized by injection of Ketamine/Xylazine according to approved protocols, under a license from the veterinary services and the Ministry of Food and Agriculture, Accra, Ghana. Primary cell cultures from embryonic kidney tissue of *E. helvum* were generated at the Kumasi Collaborative Centre for Research in Tropical Medicine, Kumasi (**Figure 1A**). After expansion of primary cells to confluent monolayers, cells were immortalized by lentiviral transduction of the SV40 large T antigen gene [19]. Primary cells could be passaged only twice, while transduced cells could be passaged continuously (>30 passages). Out of the second passage, clonal cell lines were prepared by end point dilution plating. A clonal cell line named EidNi/41.3 (**Figure 1A**) was chosen for in-depth characterization. For comparison, two clonal cell lines from the same preparation (EidNi/41.1 and EidNi/41.2), as well as the 21st passage of a mixed culture (EidNi/41), were also preserved (**Figure 1A**). Additionally, a kidney cell culture (4^th^ passage) from an adult *R. aegyptiacus* bat was prepared (data not shown) to investigate putative differences between the two related frugivorous bats. All bat cell cultures were genotyped by amplifying mitochondrial cytochrome b fragments [22] followed by a BLAST analysis. EidNi and RoNi cells showed 100% identity with cytochrome b (GenBank accession no. AB359172.1 (*E. helvum*) and JF728760.1 (*R. aegyptiacus*), data not shown).

### IFN induction

As already shown for primary cells of the *Pteropus* species, bat cells can readily secrete IFN upon virus infection or poly IC transfection [13], [14], [38], [39]. However, immortalization of cells can damage cytokine genes and signaling pathways. The reactivity of EidNi/41.3 cells to IFN stimuli was therefore tested. The induction of IFN in EidNi/41.3 cells was stimulated by two methods. First, cells were infected with RVFV 13. This virus mutant lacked its main IFN antagonist, the NSs protein. The capability of this virus to induce IFN upon infection of epithelial cells has been demonstrated [29], [40]. Second, cells were transfected with poly IC [41]. To measure the induction of IFN transcription, a real-time RT-PCR assay for the IFN-β gene was designed. To this end the IFN-β gene was amplified by nested RT-PCR from cultures of a phylogenetically representative range of bats differing in main diet and hunting strategies including the insectivorous *M. daubentonii*, *Pipistrellus spec.* (Vespertilionidae), *H. cf. caffer/ruber* (Hipposideridae),and *R. cf. landeri* (Rhinolophidae) as well as the frugivorous/nectarivorous *R. aegyptiacus* and *E. helvum* (Pteropodidae) (**Figure 1B**), using primers designed upon alignments of available IFN-β genes from horses, swine, cattle, humans, mice and rats. Real-time RT-PCR primers (see **Table S1** for GenBank no. and primer sequences) were placed in sites conserved among bats (**Figure 1C**). As shown in **Figure 1D**, EidNi/41.3 cells were readily infected by RVFV 13, and luciferase expression representing virus replication levels was comparable with that in several reference cell cultures including MEF, MA104 and Vero E6 cells. Nevertheless, the induction of the IFN-β gene in a single cycle infection experiment, at an MOI = 1, was approximately 30-fold stronger in EidNi/41.3 cells than in MEF and MA104 cells (**Figure 1E**). The induction in Vero E6 cells was not examined because of their intrinsic IFN-β gene defect [42], [43]. Interestingly, excessive IFN induction as compared to reference cells was only observed upon virus infection, but not upon poly IC transfection. It was concluded that the capability of EidNi/41.3 cells to react on natural and artificial IFN stimuli had not been affected by immortalization.

### Interferon secretion

Since cellular mRNA levels are prone to differential expression over time IFN protein secretion was analyzed next. It was tested whether EidNi/41.3 cells were able to produce and secrete effective doses of type I IFN. To this end it was necessary to compare and calibrate species-specific IFN secretion between primate, rodent, and bat cells, using an appropriate experimental model. To achieve this, a VSV-based IFN bioassay was established.

**Figure 2:**
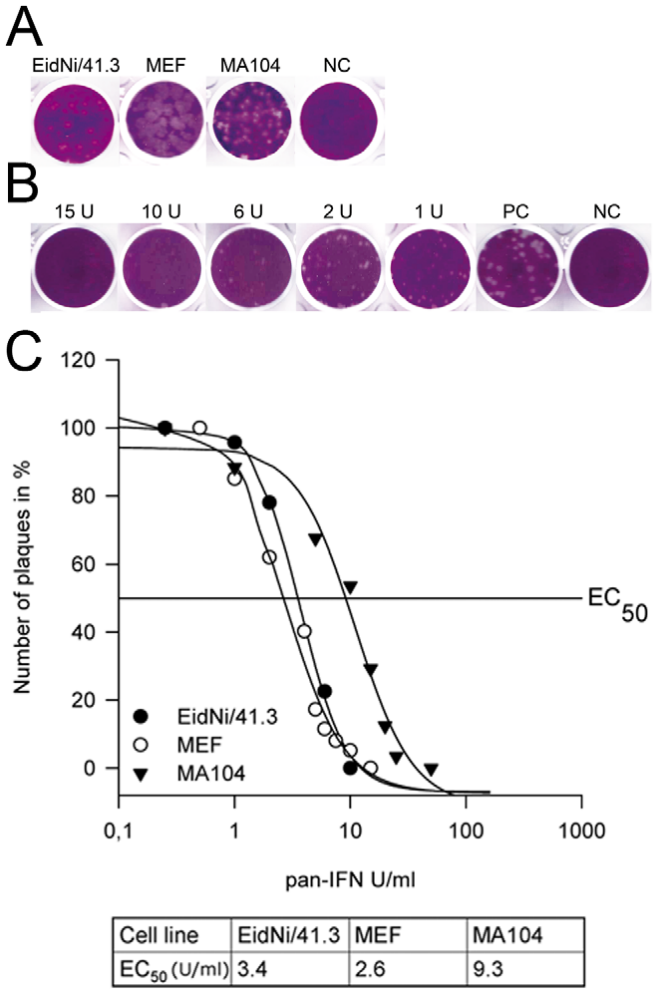
Interferon quantification and calibration by vesicular stomatitis virus (VSV) bioassay. (A) VSV plaque morphology was analyzed on the bat cell line EidNi/41.3, a rodent cell line (MEF) and a primate cell line (MA104). (B) For the VSV bioassay EidNi/41.3 cells were pre-incubated with different amounts (units per ml; U/ml) of pan-species IFN (pan-IFN). 24 h after treatment the cells were infected with VSV at an MOI of 0.025 for 1 h. After 2 days cells were fixed, stained and plaques were counted to estimate the correlation between the amounts of pan-IFN and plaques. (C) For each cell line a standard curve using different amount of pan-IFN was done and EC_50_ values were calculated. Shown are mean values of quadruplicates. Standard deviations are not shown for clarity.

VSV growth and plaque morphology was compared in all cell lines (**Figure 2A**). Countable plaques were visible after 2 days post infection (dpi). To obtain a calibration of IFN secretion across species, all cell lines were incubated with gradients of equivalent concentrations of recombinant pan-species IFN and subjected to VSV plaque reduction assays (exemplified for EidNi/41.3 in **Figure 2B**). The antiviral responses in EidNi/41.3, MEF, and MA104 cells upon equivalent doses of IFN are compared in **Figure 2C**. EC_50_ values (amount of pan-species IFN to achieve a 50% VSV plaque reduction) were calculated for all cell lines (**Figure 2C**). Using this calibration, it was possible to compare the equivalent activity levels of species-specific IFN secreted from each cell line upon stimulation.

**Figure 3:**
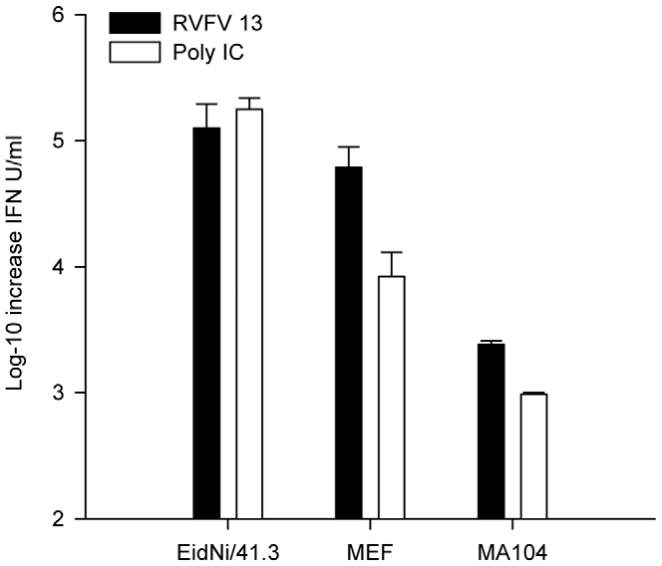
Bat cells produce high levels of secreted IFN. Cells were infected with RVFV 13 or transfected with poly IC as described before. With the help of the VSV bioassay secreted IFN was measured. Each cell line was incubated for 24 h with IFN-containing supernatants (β-propiolactone inactivated) and with pan-IFN standards diluted in medium from untreated control cells. IFN concentrations were normalized with the help of EC_50_ values as described in the Methods section.

In accordance with the IFN mRNA induction, the highest equivalent amount of bioactive secreted IFN upon RVFV 13 virus infection and poly IC transfection was measured in EidNi/41.3 cells, followed by MEF and MA104 (**Figure 3**). The observed difference in IFN mRNA induction between RVFV 13 virus and poly IC transfection (**Figure 1E**) was not seen in this case favoring the idea of differential mRNA regulation in *E. helvum* cells upon poly IC treatment.

In conclusion, EidNi/41.3 cells were capable of inducing IFN upon natural and artificial stimuli, to secrete active type I IFN, and to induce an antiviral state upon IFN stimulation.

### IFN response upon wild-type virus infection

**Figure 4:**
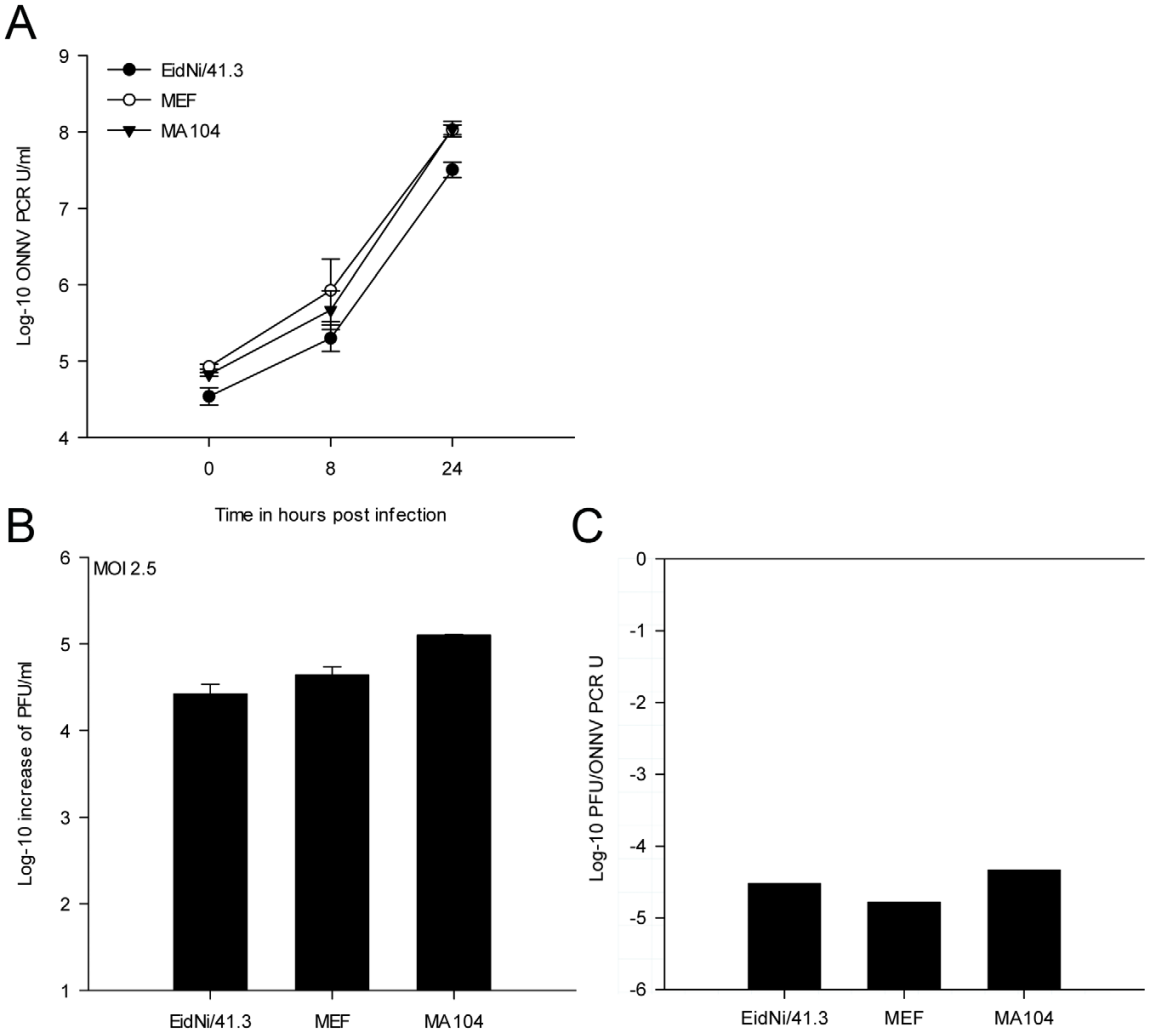
O'nyong-nyong virus (ONNV) replication in different mammalian cells using a high MOI. (A) For a synchronized infection cells were inoculated with ONNV at an MOI 2.5 and supernatants were harvested at 0, 8 and 24 h post infection (hpi). After viral RNA isolation (triplicates) the concentration was measured by ONNV specific real-time RT-PCR assay. ONNV PCR units (U) per ml were determined. The dilution end-point was defined as one PCR unit. Virus replication could be detected in all cell lines. The increase of genome equivalents per ml were approximately 1000-fold after 24 hpi. (B) Titration of supernatants showed an increase of PFU per ml (titer of inoculum was subtracted) after 24 hpi of 1000 to 10000-fold. (C) The ratio of log-10 increase PFU/ml to ONNV PCR units were comparable in all cell lines indicating an efficient particle formation.

Since *E. helvum* cells produced considerable amounts of IFN upon infection with RVFV 13 we investigated in addition the effects of infection with a non-attenuated wild-type RNA virus. The Old World Alphavirus ONNV was chosen because Alphaviruses have been detected previously in bats [44], [45] and exhibit strong induction of IFN [46]. Interestingly, Alphaviruses utilize a rather general mechanism to evade IFN, by causing a translational shutoff that affects cellular translation more than viral translation [46]–[48]. To establish a synchronized infection of ONNV, a high MOI = 2.5 was used. Virus growth was measured by an ONNV-specific real-time RT-PCR and titration of virus in supernatants 24 hpi. Virus RNA and infectious virus were detected in all cell lines (**Figures 4A and B**). Increases of infectious virus formation were about 1000-fold within 24 hpi, and specific infectivities, expressed as PFU per genome equivalent (PCR units), were highly comparable between cell cultures (**Figure 4C**).

**Figure 5:**
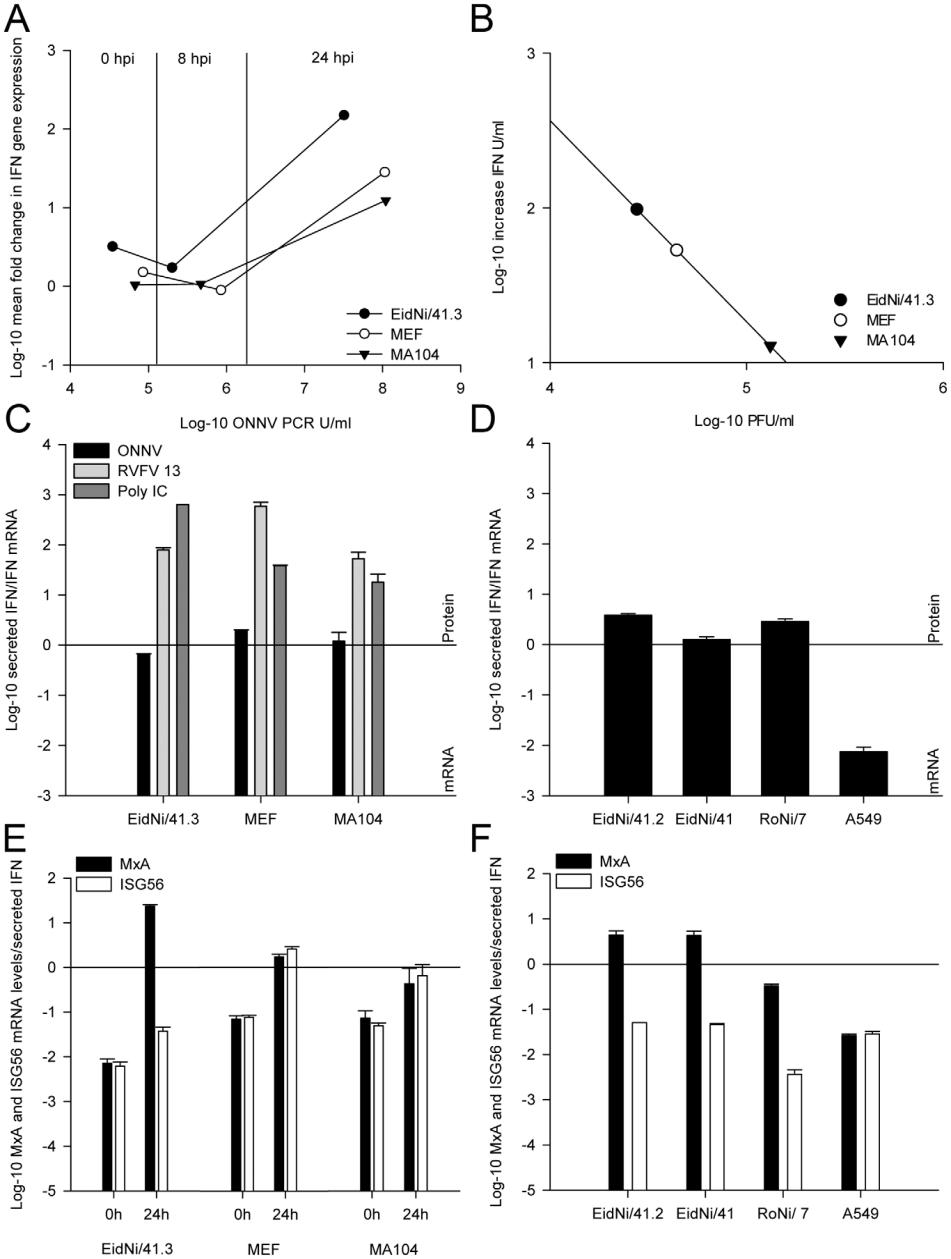
IFN-β mRNA induction but IFN protein decrease in all mammalian cells upon ONNV infection. (A) IFN-β mRNA induction was measured by species-specific real-time RT-PCR at time points 0, 8 and 24 hpi and correlated to the amount of relative ONNV genome equivalents in PCR units per ml. ONNV replication led to an induction of IFN-β mRNA 24 hpi. (B) At 24 hpi the increase of secreted IFN was correlated to ONNV plaque forming units indicating that higher virus titres led to a decreased amount of IFN in the supernatants. (C) Comparison of secreted IFN protein to IFN-β mRNA (24 hpi) after ONNV, RVFV 13 infection and poly IC transfection. ONNV replication was related to IFN protein reduction. For absolute values refer to **Figure S1**. (D) Confirmation for IFN protein reduction by testing different EidNi bat cell cultures. EidNi/41.2 (subclone), EidNi/41 (mixed cell culture), RoNi/7 (mixed cell culture from *R. aegyptiacus* kidneys) and human lung adenocarcinoma epithelial cell line (A549) showed the same phenotype. (E) Comparison of mRNA fold-induction of IFN stimulated genes (*MxA* and *ISG56*) to secreted IFN protein at time points 0 hpi and 24 hpi. Expression of ISGs was not affected by IFN protein downregulation indicating that there was no general transcriptional shutoff. (F) Confirmation by testing different cell clones and cell cultures 24 hpi indicating that *MxA* mRNA is upregulated in bat cell cultures upon ONNV infection.

To investigate if ONNV infection caused IFN induction, the amount of IFN mRNA (y-axis; for absolute values refer to **Figure S1**) was directly compared to viral RNA (x-axis) from supernatant at equivalent time points in each cell line (**Figure 5A**). Whereas no induction of IFN mRNA was detectable at 8 hpi, ONNV replication clearly induced IFN mRNA expression by 24 hpi. Comparison of secreted IFN with infectious virus production at 24 hpi revealed that increased viral titers corresponded to lower amounts of secreted IFN protein (**Figure 5B** **and S1**). EidNi/41.3 cells showed highest levels of secreted IFN but the lowest level of infectious particles, suggesting that virus replication might be limited in response to IFN.

To determine whether virus control correlated with levels of secreted IFN or merely with the induction of genes under control of the IFN promoter, the ratios of secreted IFN to IFN mRNA were determined in all cell lines. In all cells stimulated by RVFV 13 or poly IC, the ratios of secreted IFN versus IFN mRNA induction was comparable. In contrast, in all cells infected with ONNV the relative levels of secreted IFN were clearly reduced, while overall IFN mRNA induction was less affected (**Figure 5C**). This effect was independent of the cell species.

In order to exclude that the ONNV-mediated reduction of IFN protein production was cell-clone specific, another cell line named EidNi/41.2 as well as the original mixed cell culture EidNi/41 was analyzed, essentially showing the same reduction of relative IFN secretion upon ONNV infection (**Figure 5D**). Similar effects were also seen in a mixed cell culture generated from the related flying fox *R. aegyptiacus* (RoNi/7). A human lung adenocarcinoma epithelial cell line (A549) showed an even higher decrease of IFN secretion than MA104 and MEF (**Figure 5D**).

Alphaviral induction of a transcriptional and/or translational shutoff affecting mainly the cellular but not the viral protein production has been described in other cells [46]. While our results suggested similar effects to be in place in *E. helvum* and *R. aegyptiacus cells*, control of ONNV replication seemed to be relatively more efficient in both applied bat cells. One reason might be differences in the capability of bat cells to react on IFN signaling. To investigate this, the mRNA abundance of two different ISGs (MxA and ISG56) as well as two reference genes (actin-β and TBP) was analyzed and compared to the amounts of secreted IFN at 0 and 24 hpi. Whereas the expression of MxA is strictly IFN signaling-dependent, ISG56 can also be directly activated in the absence of secreted IFN and without involvement of the JAK/STAT pathway [49], [50]. As shown in **Figure 5E**, the ratios of MxA and ISG56 mRNA to secreted IFN showed an approximately 10-fold increase 24 hpi. Thus, transcription did not seem to be generally blocked after ONNV infection in all cells.

In bat cells (EidNi/41.3) but not in murine or primate cells, the level of MxA mRNA induction in relation to IFN in supernatant was more than 1000-fold higher compared to the same ratio for ISG56. This was also the case in different clonal and mixed *E. helvum* kidney cells (EidNi/41.2 and EidNi/41), as well as in *R. aegyptiacus* kidney (RoNi/7) cells (**Figure 5F**), suggesting that IFN signaling-dependent induction of ISGs may be highly efficient in *E. helvum* and *R. aegyptiacus* bat cells. In our experiments this explained an apparently superior control of ONNV replication in both applied bat cells.

**Figure 6:**
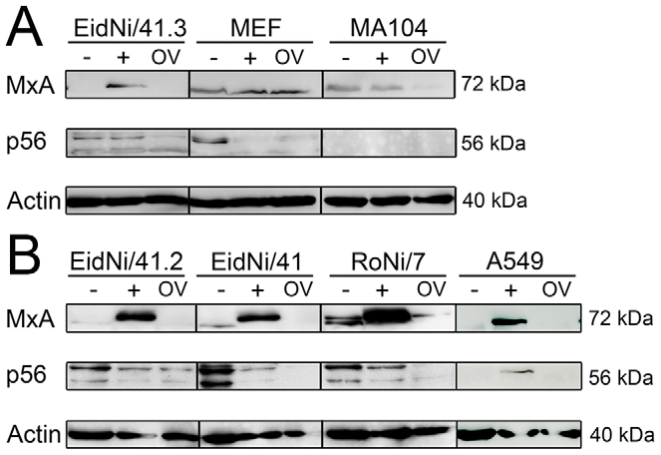
ONNV infection ablates the expression of IFN stimulated genes. (A) Cells were either left untreated (−) or infected with RVFV 13 (+) or ONNV (OV). After 24 h proteins were extracted from cells. Same amount of proteins were subjected to SDS-PAGE followed by a Western blot analysis. Mouse-anti-Mx1/2/3 (MxA), goat-anti-IFIT1/ISG56 (p56) and mouse-anti-actin immunoglobulins were applied at dilutions 1∶1000 followed by a peroxidase labeled goat-anti-mouse or rabbit-anti-goat secondary antibody (1∶20000). In all cell lines infection with ONNV did not induce the expression of MxA and p56 and was less or comparable to untreated cells. (B) To exclude cell clone specific effects additional EidNi and RoNi bat cell cultures were included (EidNi/41.2; EidNi/41 and RoNi/7) as well as a human A549 cell line.

To confirm alphaviral translational shutoff in bat cells, protein expression of MxA, p56 and actin was determined by Western blot analysis 24 hpi. As depicted in **Figure 6**, RVFV 13 infection clearly caused higher or similar (MA104 cells) expression of MxA in cells from all species while expression of p56 was hardly increased over levels seen in uninfected cells. In contrast, ONNV infection resulted in a reduced expression of MxA and p56 proteins. This effect was generally more pronounced for MxA than for p56, suggesting that IFN-dependent signaling rather than JAK/STAT-independent induction were affected (**Figure 6**). Interestingly, this effect was observed in all applied bat cell lines but not in MEF (**Figure 6A**). In summary, while *E. helvum* and *R. aegyptiacus* bat cells showed particularly efficient induction of MxA mRNA, the production of this highly active antiviral protein was antagonized by ONNV in those bat cells to a larger extent than in cells from rodents or primates.

**Figure 7:**
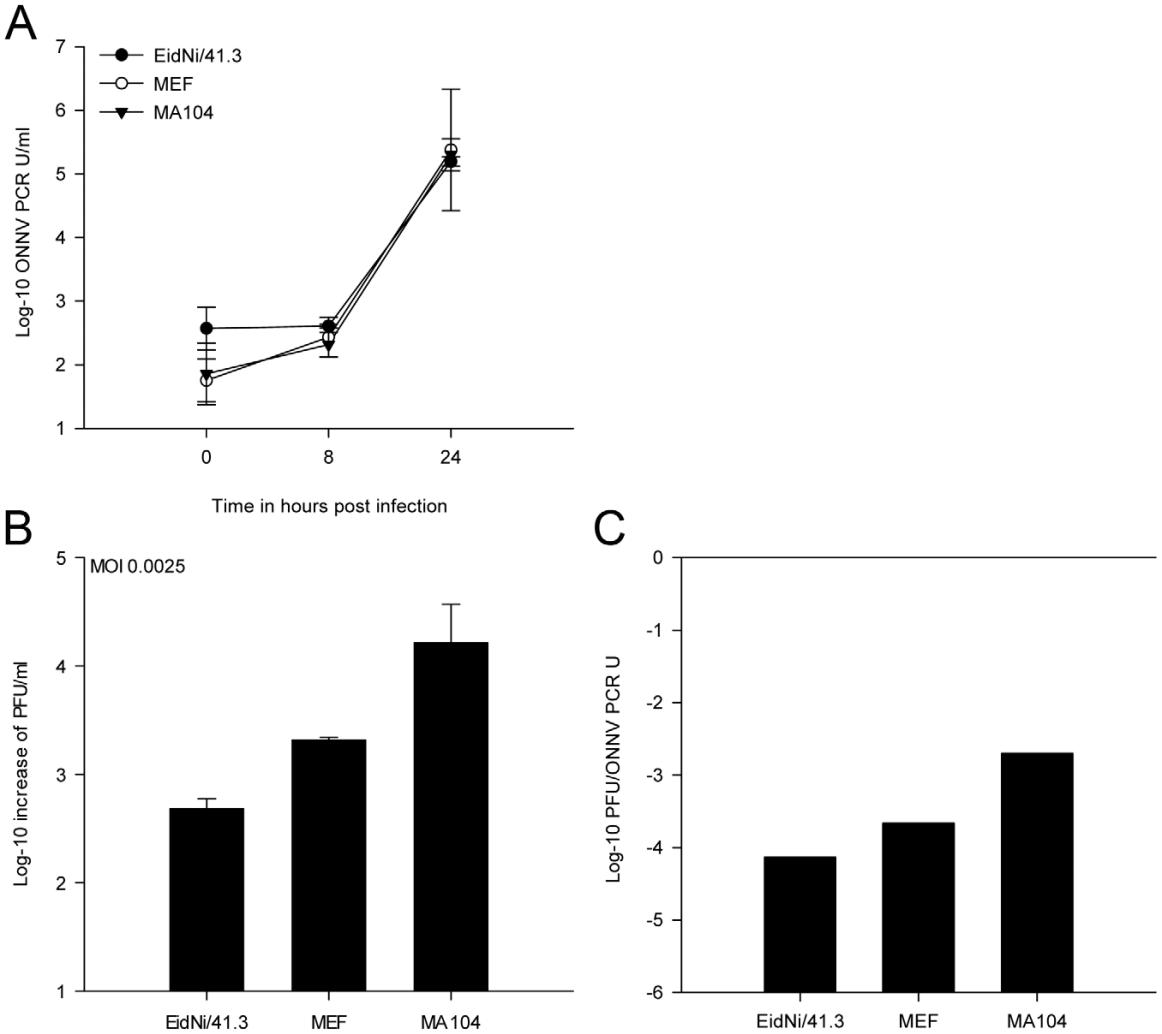
Efficient ONNV replication upon infection at a low MOI. (A) In order to analyze if insufficient viral replication led to a delayed or ablated IFN production in cells a growth kinetic at low MOI was performed (MOI 0.0025). Cells were inoculated with ONNV and supernatants were analyzed at 0, 8 and 24 hpi by real-time RT-PCR. Virus replication could be detected in all cell lines. (B) Supernatants were titrated after 24 hpi confirming the PCR results. (C) PFU to ONNV PCR unit (U) ratio after infection of cells with ONNV at an MOI of 0.0025. In MA104 and MEF cells the infection at low MOI resulted in a higher PFU to genome equivalent ratio compared with infection at high MOI.

Finally, it had to be excluded that the observed reduction of IFN and ISG protein levels might be due to attenuation of virus replication. Alphaviruses have been shown to acquire mutations in the 5′UTR [51], [52], nsP3 [53], or nsP2 genes [54], which resulted in low level replication and enabled virus persistence without IFN-dependent elimination. The observed increase of IFN mRNA upon ONNV infection (**Figure 5A**) and viral titers of up to 10^5^ PFU per ml (**Figure 4B**) would in fact speak against this hypothesis. However, to exclude attenuated replication in bat cells we analyzed virus growth at a very low MOI of 0.0025. Virus replication was monitored by real-time RT-PCR at time point 0, 8 and 24 hpi (**Figure 7A**) and viral supernatants were titrated after 24 h (**Figure 7B**). Additionally, the specific infectivity of virus supernatants was compared 24 hpi (**Figure 7C**). All cell cultures supported virus growth efficiently, despite 1000-fold lower MOIs applied in this experiment. The specific infectivity was higher compared to the infection at MOI = 2.5, suggesting an efficient infectious particle formation in all cells at low MOI. Whereas MA104 primate cells yielded a 10 to 100-fold higher specific infectivity compared to the high MOI experiment, EidNi/41.3 and MEF had similar ratios at both doses. Taken together the highly productive virus formation at a low MOI suggested that decreased IFN and ISG protein levels were generally not due to attenuation of replication.

In conclusion, this study is the first to directly compare IFN induction, secretion and signaling in African fruit bat cell lines with that in other mammalian cell lines. We show that these bat cells were fully capable of reacting to viral and artificial IFN stimuli. Our bat cells showed strikingly high IFN mRNA induction, efficient IFN protein secretion, and evidence of highly efficient ISG induction. ONNV exhibited typical strong IFN induction but evaded the IFN response by translational rather than transcriptional shutoff, as typical in Alphavirus infection of mammalian cells. These well-characterized, highly interferon-competent cell lines will enable comparative research on zoonotic, bat-borne viruses in order to model mechanisms of viral maintenance and emergence in bat reservoirs.

## Supporting Information

Figure S1 **Detection of IFN-β mRNA induction and species-specific IFN in different cells after infection with ONNV.** (A) Cells were infected with ONNV (MOI 2.5) and IFN-β mRNA induction was quantified by real-time RT-PCR. (B) With the help of a VSV bioassay an increase of secreted IFN was measured. Each cell line was incubated for 24 h with IFN containing supernatants (β-propiolactone inactivated) or with pan-IFN standards diluted in medium from negative control cells. IFN concentrations were normalized with the help of EC_50_ values as described in the Methods section. In EidNi/41.3 bat cells IFN-β mRNA induction and increase of IFN protein secretion were both approximately 100-fold. MA104 and MEF cells experienced a 10 to 50-fold increase of IFN-β mRNA induction and IFN protein. (C) Confirmation of low level IFN secretion upon ONNV infection in different cell cultures and clones.
(TIF)

Table S1 **Oligonucleotides for real-time RT-PCRs and genotyping.** Overview of all amplified gene sequences and designed oligonucleotides for species-specific real-time RT-PCRs.
(DOC)
