# Supplementary figures and images for: Type I Interferon Reaction to Viral Infection in Interferon-Competent, Immortalized Cell Lines from the African Fruit Bat Eidolon helvum
Source: PLoS One. 2011 Nov 30;6(11):e28131. doi: 10.1371/journal.pone.0028131 (PMC3227611; doi:10.1371/journal.pone.0028131)

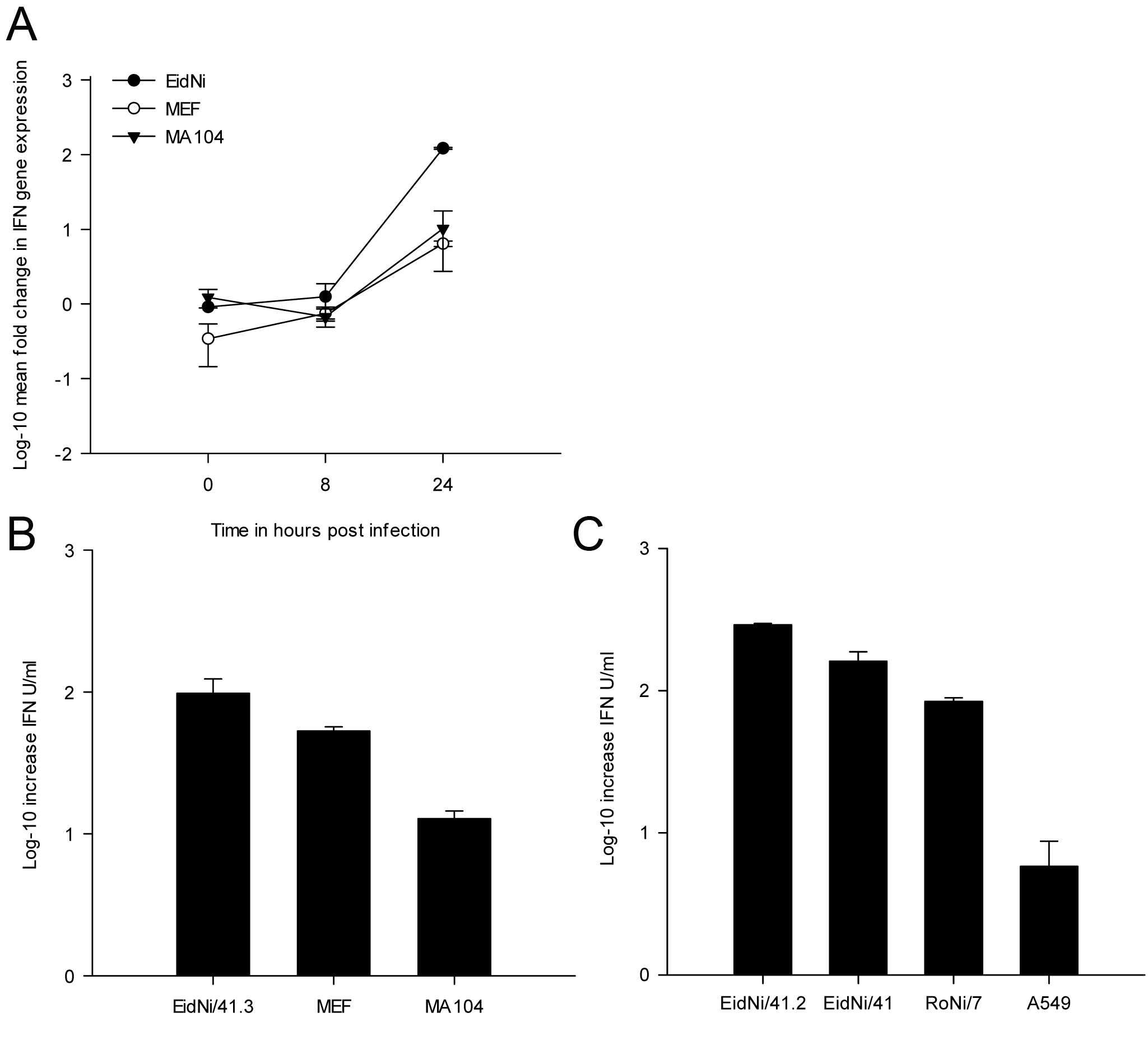

Supplement: Figure S1 — Detection of IFN-β mRNA induction and species-specific IFN in different cells after infection with ONNV. (A) Cells were infected with ONNV (MOI 2.5) and IFN-β mRNA induction was quantified by real-time RT-PCR. (B) With the help of a VSV bioassay an increase of secreted IFN was measured. Each cell line was incubated for 24 h with IFN containing supernatants (β-propiolactone inactivated) or with pan-IFN standards diluted in medium from negative control cells. IFN concentrations were normalized with the help of EC50 values as described in the Methods section. In EidNi/41.3 bat cells IFN-β mRNA induction and increase of IFN protein secretion were both approximately 100-fold. MA104 and MEF cells experienced a 10 to 50-fold increase of IFN-β mRNA induction and IFN protein. (C) Confirmation of low level IFN secretion upon ONNV infection in different cell cultures and clones. (TIF) [file pone.0028131.s001.tif]
