# Supplementary material for: Type I Interferon Reaction to Viral Infection in Interferon-Competent, Immortalized Cell Lines from the African Fruit Bat Eidolon helvum
Source: PLoS One. 2011 Nov 30;6(11):e28131. doi: 10.1371/journal.pone.0028131 (PMC3227611; doi:10.1371/journal.pone.0028131)
Supplement: Table S1 — Oligonucleotides for real-time RT-PCRs and genotyping. Overview of all amplified gene sequences and designed oligonucleotides for species-specific real-time RT-PCRs. (DOC) [file pone.0028131.s002.doc]

| **Assay** | **Name** | **Primer [5´-3´]** | **GenBank ID/Reference** |
| --- | --- | --- | --- |
| **Actin-β (ACTB)** | | | |
| **bat** | qRT-bACTB_for | GGCTCCCAGCACAATGAAGA | JN613815  JN613816  JN613817  JN613818 |
|  | qRT-bACTB_prb | 6-FAM-CAAGATCATTGCGCCCCCTGAGC-BHQ-1 |  |
|  | qRT-bACTB_rev | GGA GCC GCC GAT CCA |  |
| **mouse** | mActb_for | AAATCGTGCGTGACATCAAAGA | NM_008331.3 |
|  | mActb_prb | 6-FAM-ATGGCCACTGCCGCATCCTCTTC-BHQ-1 |  |
|  | mActb_rev | GAACCGCTCGTTGCCAATAG |  |
| **human** | qRT_hpmACTB_fwd | GAGGCATCCTCACCCTGAAG | NM_001101.3  NM_001009945.1  NM_001033084.1 |
|  | qRT_hpmACTB_prb | 6-FAM-CCCCATCGAGCACGGCATCG-BHQ-1 |  |
|  | qRT_hpmACTB_rev | TCCATGTCGTCCCAGTTGGT |  |
| **Interferon-β (IFNB1)** | | | |
| **bat** | bIFNB1-fwd | CAGCTATTTCCATGAGCTACAACTTG | JN613819  JN613820  JN613821  JN613822 |
|  | bIFNB1-prb | 6-FAM-TCGATTCCAACAAAGAAGCAGCAATTTAGC-BHQ-1 |  |
|  | bIFNB1-rev | TTAACTGCCACAGGAGCTTCAG |  |
| **mouse** | mIfnb1_for | AGAAAGGACGAACATTCGGAAAT | NM_010510.1 |
|  | mIfnb1_prb | 6-FAM-ATGGAAAGATCAACCTCACCTACAGGGCG-BHQ-1 |  |
|  | mIfnb1_rev | GGATGGCAAAGGCAGTGTAACT |  |
| **human** | IFN F | gAACTTTgACATCCCTgAggAgATT |  |
|  | IFN P | 6-FAM-CAGCAGTTCCAGAAGGAGGACGCC-BHQ-1 |  |
|  | IFN R | ggAgCATCTCATAgATggTCAATg |  |
| **Interferon stimulated gene 56 (ISG56)/Interferon-induced protein with tetratricopeptide repeats 1 (IFIT1)** | | | |
| **bat** | qRT-bISG56_for | TTGAAGAAGCTCTGGCCAACA | JN613823  JN613824  JN613825  JN613826 |
|  | qRT-bISG56_prb | 6-FAM-ACAGACCTACGTCTTTCGATATGCAGCCA-BHQ-1 |  |
|  | qRT-bISG56_rev | GCG CCT TTT CTT CGG TAA AA |  |
| **mouse** | mIsg56_for | TGCCTGGCTGCATTACCA | NM_008331.3 |
|  | mIsg56_prb | 6-FAM-TTGGCAGAAGCCCAGATCTACCTGGAC-BHQ-1 |  |
|  | mIsg56_rev | TGAAAATTCCTTGCACACCTTCT |  |
| **human** | ISG56 F | CCTGGAGTACTATGAGCGGGC |  |
|  | ISG56 P | 6-FAM-ACAGAGTTCTCAAAGTCAGCAGCCAGTCTCAGT-BHQ-1 |  |
|  | ISG56 R | TGGGTGCCTAAGGACCTTGTC |  |
| **Myxovirus (influenza virus) resistance 1 (MX1/MxA)/interferon-inducible protein p78** | | | |
| **bat** | qRT-bMxA_for | CCGATCTGACCCTCATCGA | JN613827  JN613828  JN613829  JN613830 |
|  | qRT-bMxA_prb | 6-FAM-TGGGCAACCAGCCCCAGGAC-BHQ-1 |  |
|  | qRT-bMxA_rev | ATG GTC TGC TGC CTC TGG AT |  |
| **mouse** | mMxA1_for | TGAATAATCTGTGCAGGCACTATGA | NM_010846.1 |
|  | mMxA1_prb | 6-FAM-TGCGGCCCTGTATTGACCTCATCG-BHQ-1 |  |
|  | mMxA1_rev | CTGAACTCTGGTCCCCAATGA |  |
| **human** | MxA F | TTCAGCACCTGATGGCCTATC |  |
|  | MxA P | 6-FAM-CAGGAGGCCAGCAAGCGCCATC-BHQ-1 |  |
|  | MxA R | TGGATGATCAAAGGGATGTGG |  |
| **TATA box binding protein (TBP)** | | | |
| **bat** | qRT-bTBP_for | TTGCTGCTGTGATCATGAGAATT | JN613831  JN613832  JN613833 |
|  | qRT_bTBP_prb | 6-FAM-CCCGGACCACGGCCCTGA-BHQ-1 |  |
|  | qRT_bTBP_rev | CCCGGACCACGGCCCTGA |  |
| **mouse** | mTbp_for | TGTACCGCAGCTTCAAAATATTGT | NM_013684.3 |
|  | mTbp_prb | 6-FAM-TCCCAAGCGATTTGCTGCAGTCATC-BHQ-1 |  |
|  | mTbp_rev | AGTTGTCCGTGGCTCTCTTATTCT |  |
| **human** | qRT_hTBP_fwd | GCTGCGGTAATCATGAGGATAAG | NM_003194.4 |
|  | qRT_hTBP_prb | 6-FAM-AGCCACGAACCACGGCACTGATTTT-BHQ-1 |  |
|  | qRT_hTBP_rev | TGCACACCATTTTCCCAGAA |  |
| **O´nyong-nyong virus (ONNV), Gulu strain** | | | |
|  | ONNV-S | tgatccagactcaaccattct | M20303.1 |
|  | ONNV-P | 6-FAM-accagctaggaggatgatgtctga-bBQ-1 |  |
|  | ONNV-AS | ggcagacgcagtggtattttct |  |
| **Simian virus 5 (SV5)/Parainfluenza virus 5 (PIV5)** | | | |
|  | SV5-F | CGTGGGGGATCCCTTCA | [AF052755](http://www.ncbi.nlm.nih.gov/nuccore/AF052755) |
|  | SV5-P | 6-FAM-CCCACCAGCAGATACCAGTCAATTTGATC-BHQ-1 |  |
|  | SV5-R | CCACCTCTGGGTGATACAATGA |  |
| **Genotyping (mitochondrial cytochrome b)** | | | |
|  | L14724 | CGAAGCTTGATATGAAAAACCATCGTTG |  |
|  | H15149 | AACTGCAGCCCCTCAGAATGATATTTGTCCTCA |  |
